# Novel compound heterozygous pathogenic variants in *ASCC1* in a Chinese patient with spinal muscular atrophy with congenital bone fractures 2 : Evidence supporting a "Definitive" gene‐disease relationship

**DOI:** 10.1002/mgg3.1212

**Published:** 2020-03-11

**Authors:** Weiliang Lu, Mingxing Liang, Jiasun Su, Jin Wang, Lingxiao Li, Shujie Zhang, Zailong Qin, Limei Huang, Yingchi Lu, Shang Yi, Sheng Yi, BoBo Xie, Haiyang Zheng, Jingsi Luo, Xiaoyan Gao, Yiping Shen

**Affiliations:** ^1^ Genetic and Metabolic Central Laboratory Birth Defect Prevention Research Institute Maternal and Child Health Hospital Children’s Hospital of Guangxi Zhuang Autonomous Region Nanning China; ^2^ Department of Neonatology Maternal and Child Health Hospital of Guangxi Zhuang Autonomous Region Nanning China; ^3^ Department of Medical Genetics and Molecular Diagnostic Laboratory Shanghai Children’s Medical Center Shanghai Jiao Tong University School of Medicine Shanghai China; ^4^ Division of Genetics and Genomics Boston Children’s Hospital Boston MA USA; ^5^ Department of Neurology Harvard Medical School Boston MA USA

**Keywords:** *ASCC1*, compound heterozygous, exome sequencing, gene curation

## Abstract

**Background:**

A very limited spectrum of *ASCC1* pathogenic variants had been reported in six (mostly consanguineous) families with spinal muscular atrophy with congenital bone fractures 2 [OMIM #616867] since 2016.

**Methods:**

A proband from a non‐consanguineous Chinese family presented with neonatal severe hypotonia, respiratory distress, muscle weakness, and atrophy, as well as congenital bone fractures was performed by exome sequencing.

**Results:**

A compound heterozygosity of a nonsense (c.932C>G,p.Ser311Ter) and an exon 5 deletion in *ASCC1* segregating with phenotypes was detected, both variants are novel and pathogenic. Since *ASCC1* is a relatively new disease gene, we performed the gene curation by ClinGen SOP. The existing evidence is sufficient to support a "Definitive" level of disease‐gene relationship.

**Conclusion:**

This case report expended the mutation spectrum of *ASCC1* and support the notion that this novel disease also occurs in outbreed populations and this is a rare disease but may still be underdiagnosed due to its perinatal lethal outcomes.

## Graphical abstract

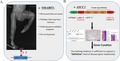

## 1 INTRODUCTION

*ASCC1* encodes a subunit of the activating signal cointegrator 1 (ASC‐1) complex. The ASC‐1 complex is a transcriptional coactivator that plays an important role in gene transactivation by multiple transcription factors and *ASCC1* is an essential component for AP‐1 transactivation in vivo (Jung et al., 2002). The previous study showed that *ASCC1* pathogenic variants were associated with Barrett esophagus and esophageal adenocarcinoma (Orloff et al., 2011). Recently, using WES, *ASCC1* pathogenic variants were reported to cause bone fractures in neonates with lethal outcomes. Knierim et al. (2016) identified a homozygous frameshift variant (c.157dupG, p.Glu53Glyfs*19) in *ASCC1* in a patient with prenatal‐onset spinal muscular atrophy (SMA), multiple congenital contractures (arthrogryposis multiplex congenita), respiratory distress, and congenital bone fractures. The same variant at homozygous status was identified by Oliveira, Martins, Pinto Leite, Sousa, and Santos (2017) in a patient with severe neonatal hypotonia, lack of spontaneous movements, microretrognathia, and arthrogryposis, bilateral femoral fractures and thin, gracile ribs. Three additional families were reported by Böhm et al. (2019) with prenatal‐onset muscle weakness with arthrogryposis and congenital bone fractures, and Giuffrida et al. (2019) also reported an exonic microdeletion (exons 6–9 of the *ASCC1* gene) and a nucleotide variant (c.1027C>T/p.Arg343*) in a new case of SMA with congenital bone fractures 2 who was diagnosed in a stillbirth. Two of the families were consanguineous and five affected individuals had the same homozygous variant. Only two families showed a compound heterozygous variant including the nonsense variant (c.667C>T/p.Glu223*) and the recurrent frameshift variant (Böhm et al., 2019) as well as the exonic deletion (exons 6–9) and the nonsense variant (c.1027C>T(p.Arg343*; Giuffrida et al., 2019). Thus, a total of 11 patients from six independent families had been reported to be similarly affected. A total of five variants were uncovered so far and the recurrent frameshift variant occurred in six patients from three families. The narrow mutation spectrum of *ASCC1* in patients mostly from consanguineous families limited our understanding of this severe condition. Here, we report the first Chinese case and revealed two novel pathogenic variants, implicating the condition may be more widespread.

## 2 MATERIALS AND METHODS

### 2.1 Human subjects

This work was approved by the Ethics Committee of Guangxi Maternal and Child Health Hospital. Written informed consent was obtained from the family.

### 2.2 Whole‐exome sequencing

Genomic DNA was extracted from the peripheral blood samples. Target capture was done using an Agilent SureSelect Human All Exon kit (Agilent) according to the manufacturer's protocols, and sequencing was performed on an Illumina HiSeq2000 (Illumina). The Genome Analysis Toolkit (GATK) was employed for reads alignment and variant detection. The TGex software (LifeMap Sciences) was used to prioritize the SNVs and indels. A reads‐depth based custom pipeline using a control dataset as reference was used for CNV detection, CNV was further visually inspected using integrative genomics viewer. The variant pathogenicity was assessed according to ACMG/AMP guidelines (Richards et al., 2015).

### 2.3 ASCC1 targeted variant analysis

Sanger sequencing was carried out in the patient and in parental samples to confirm the nonsense variant identified by WES. The variant was described by the accession number NM_001198798.2 (ENST00000394919) for *ASCC1*. To confirm deletions involving exonic sequences, we designed one pair of MLPA probes for 3–8 exons. Since exon 5 is relatively large, we designed two pairs of probes for exon 5 (Table 1).

**Table 1:** The MLPA probe sequences

Exon (Ex)	Target‐speific hybridization sequences (5′−3′)	GC (%)	*T*m (°C)	Target sequence length (bp)	Total amplicom size (bp)
*ASCC1* EX3	C TCCATGGAGT GTGCTGATGA GCCCTGTGAT GCCTACGAGG TGGAGCAGAC CCCACAA	55 63	79.95 80.26	58	100
*ASCC1* EX4	GAGAGGGGAC ACTAGGAAGA AAATAGAAAT GGAGA CCAAA ACTTCTATTAGCATTCCTAA ACCTGGACAAGACGGGGA	43 44	74.26 80.08	78	120
*ASCC1* EX5a	CACTGG CCAGCATCGA AATGGTGTAATTTCA GCCCG AACACGGATT GATGTTCTTT TGGACACT	45 48	76.41 79.21	64	108
*ASCC1* EX5b	C CTCAATGAAG TTGAGGTTCAGGAAGGATTC CTGA GATTCC AGGAGGAAGT ACTGGCGAAG TGCTCCATG	46 54	78.03 81.22	70	112
*ASCC1* EX6	CATCTAACTA TTGGGATGTTGGTGCTTTTG AGTGAGGAAG AGATCCAGCA GACATGTGAG ATGCTACAGCAGTG	42 50	77.61 77.10	74	116
*ASCC1* EX7	GATATTTC TGGGGGTAAA CCCCTAGAAGTGGAGATGGCAG GGATAGAA TACATGAATG ATGATCCTGG CATGGTGGATGTTC	50 43	80.92 79.50	82	124
*ASCC1* EX8	GC TACAAGAATT AGTTGATCGAGTGCTGGAAC GTTTTCAGGC ATCTGGACTA ATAGTGAAAG AGTGGAATAGTGTGAAACTGCATGC	45 40	79.83 76.86	87	129

The MLPA was performed using the MLPA kit (P200‐Reference‐1‐B1; MRC‐Holland) according to the manufacturer's instructions.

## 3 RESULTS

### 3.1 Clinical description

**Figure 1:**
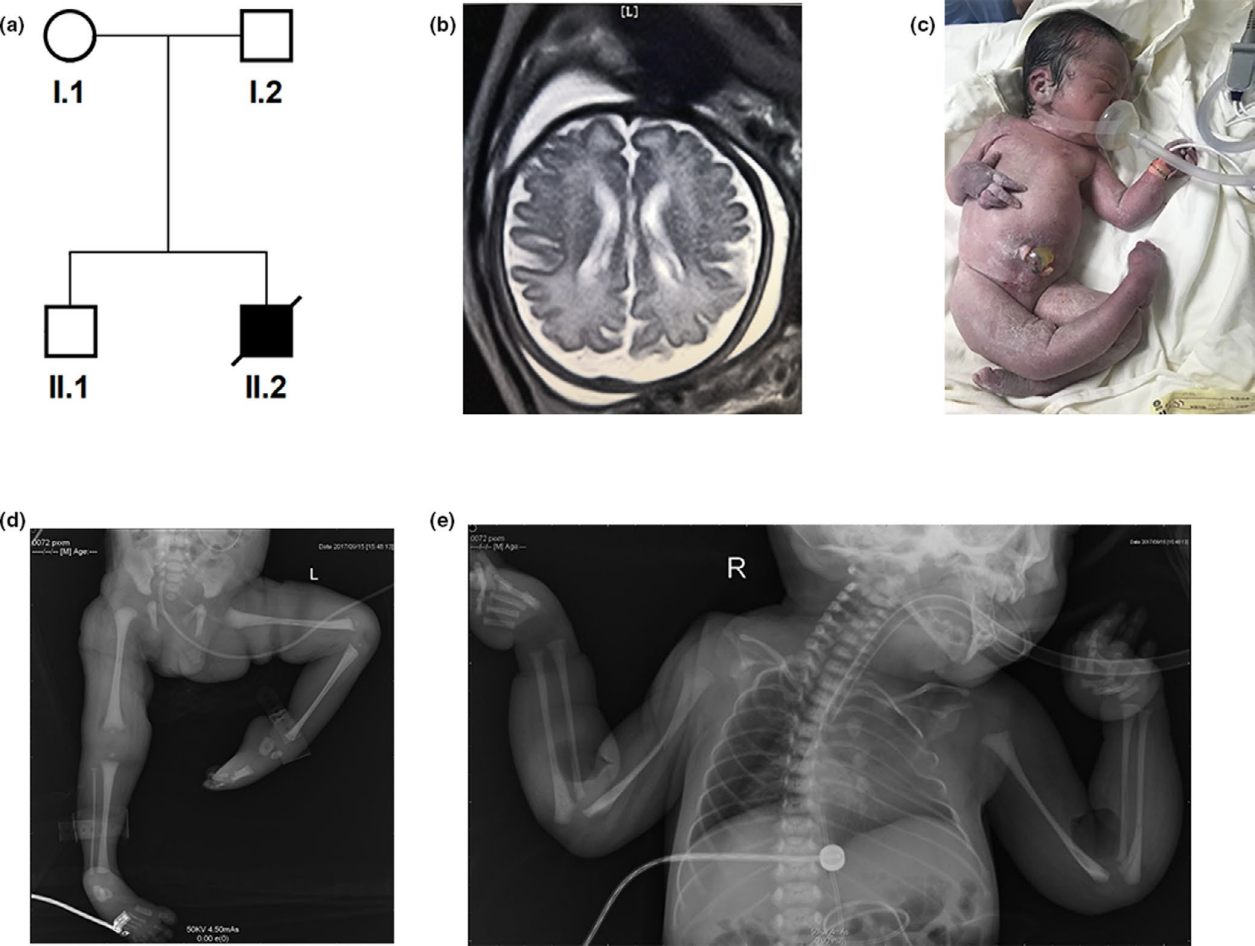
Family pedigree and patient's main clinical features. (a) Pedigree of the family compatible with an autosomal recessive inheritance pattern. (b) The MRI images of affected children II.2 (a). (c) Myopathic appearance, including congenital multiple deformity, neonatal respiratory distress, and talipes equinovarus. (d, e) The malformation of lower limb and the bilateral congenital femoral fractures

The proband was born at 37 weeks of gestation to a healthy mother (G2P2) from a non‐consanguineous family (Figure 1a). At 32 weeks of gestation, fetal ultrasound examination revealed an abnormal posture of upper limbs and club feet. The brain MRI indicated a mild bilateral lateral ventricle dilation with the left and right lateral ventricle posterior horn of approximately 1.26 and 1.12 cm in size, respectively (Figure 1b). The delivery was uneventful with APGAR scores 5, 8, 8 at 1, 3 and 5 min, respectively. The neonate presented with pervasive edema, hypotonia, talipes equinovarus and humeral fractures (Figure 1c–e). He had respiratory distress accompanied by pneumonia, coagulation abnormality, and cryptorchidism. His ears were posteriorly rotated and low‐set. He passed away on day 2 after birth. Detailed clinical data are shown in Table 2.

**Table 2:** Clinical features of the patient reported in this work and comparison with published cases with ASC‐1‐related neuromuscular diseases

	Knierim et al. (family 1)	Olivera et al. (family 2)	Böhm et al. (family 3)	Böhm et al. (family 4)	Böhm et al. (family 5)	Giuffrida et al. (family 6)	This case
Number of patients (*n*)	2	2	2	1	3	1	1
Country	Turkey	Portugal	Tunisia	Morocco	Sri Lanka	Italy	China
Gender	Female	Male/Female	Male	Female	Male/Female	Female	Male
Locus	*ASCC1*	*ASCC1*	*ASCC1*	*ASCC1*	*ASCC1*	*ASCC1*	*ASCC1*
Pathogenic variant	c.157dupG (p.Glu53Glyfs*19) homo	c.157dupG (p.Glu53Glyfs*19) homo	c.157dupG (p.Glu53fs19*) homo	c.157dupG (p.Glu53fs19*) c.466C>T (p.Arg156*) compound heterozygous	c.667C>T (p.Glu223*) homo	arr[GRCh37] 10q22.1(73873192_73936961)x1; c.1027C>T(p.Arg343*) compound heterozygous	c.932C>G (p.Ser311Ter); Exon 5del compound heterozygous
Reduced/absent fetal movements	Y	Y	Y	U	Y	U	Y
Poly/hydramnios	Y	N	U	U	U	Y	N
Oligohydramnios	N	N	U	U	U	N	N
Premature delivery (<37 weeks)	Y	N	U	N	U	Y	N
Neonatal hypotonia	Y	Y	Y	Y	Y	U	Y
Neonatal respiratory distress	Y	Y	Y	Y	Y	Y	Y
Congenital bone fractures	Y	Y	Y	Y	Y	Y	Y
Joint contractures	Y	Y	Y	Y	Y	Y	Y
Muscle weakness and atrophy	Y	Y	Y	Y	Y	Y	Y
Cardiomyopathy	N	N	N	N	N	Y	N
Skin changes	U	N	U	U	U	U	Y
Brain imaging	Abnormal cortical gyration (MRI)	N (transfontanel ultrasonography)	NP	NP	N	NP	Y (lateral ventricle dilatation, mild)
Skeletal muscle histology	Fiber size variation and atrophy. Type 1 fiber grouping	Fiber atrophy (limited analysis, in the context of autopsy)	Fiber size variability, oxidative rims	Fiber size variability, oxidative rims	Fiber size variability, oxidative rims, type I fiber predominance	Increased height of the vertebral bodies, abnormal maturation of the sternum,advanced maturation of iliac bones	NP
Severity	U (died between 2 weeks and 16 months of life	Died within a few days of life	Deceased shortly after birth	Deceased at 13 days	Deceased shortly after birth	Stillbirth	Deceased at 2 days

Abbreviations: N, no; NA, not applicable; NP, not performed; U, unknown; Y, yes.

### 3.2 Mutation analysis

**Figure 2:**
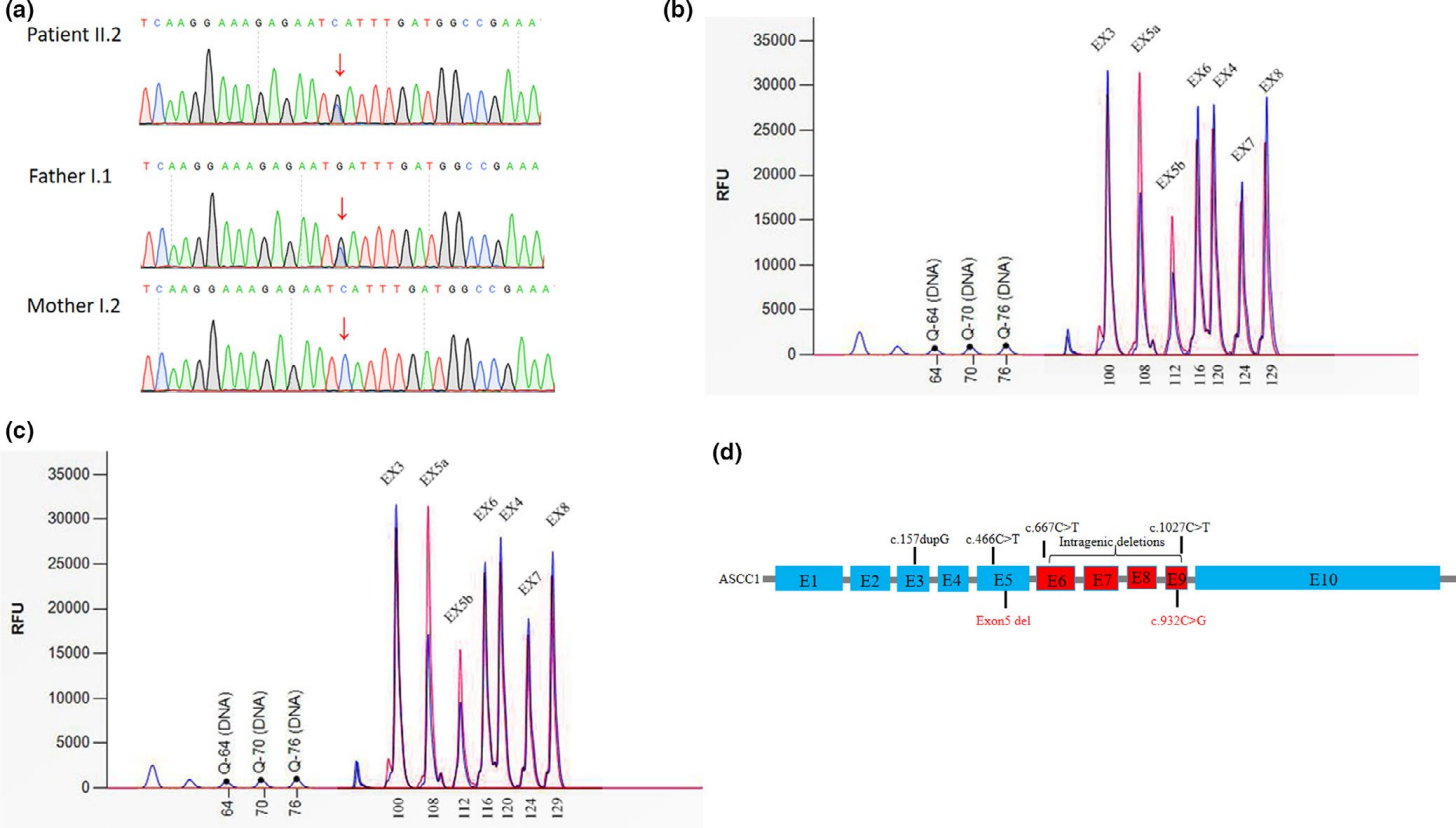
Identification of NM_001198798.2:c.932C>G and Exon 5 del variants in *ASCC1* gene. (a) Confirmation of variant by Sanger sequencing in the patient and in both parents. (b) A deletion mutation in exon 5 was detected in the patient by using MLPA, and the mother was the carrier of this variant (c). The red peak represents the control that carried a complete *ASCC1* gene, the blue peak represents the deletion of exon 5 in the proband and his mother; the *X*‐axis represents the amplicons of 3–8 exons by MLPA. (d) Exon structure of the *ASCC1* gene and distribution of the mutations. The published mutations are depicted in black, the new *ASCC1* mutations in our patient are highlighted in red

A heterozygous nonsense variant, c.932C>G/p.Ser311Ter in *ASCC1*was detected in the proband and the father. It is predicted to lead to nonsense‐mediated decay. (Figure 2a). We also detected an intragenic deletion involving exon 5 in the proband (Figure 2b) which presumably resulted in an out‐of‐frame change. The mother was the carrier of the deletion (Figure 2c). Both variants had not been previously reported. Both are null variants and are pathogenic according to the ACMG/AMP guidelines (Richards et al., 2015).

## 4 DISCUSSION

*ASCC1*(OMIM: 616867) contains 10 coding exons. It encodes a protein of the ASC‐1 cointegrator complex that mediates the interaction of transcription factors with the basal transcription machinery to modulate gene expression (Jung et al., 2002). More recently, *ASCC1* was suggested to be a ribonucleoprotein complex involved in transcriptional coactivation of a wide range of genes and in RNA processing. Knierim et al. showed that *ASCC1* mutation would downregulate genes associated with neurogenesis, neuronal migration, and pathfinding, including *SERPINF1, DAB1, SEMA3D*, and *SEMA3A*, as well as the genes which associated with bone development such as *TNFRSF11B, RASSF2*, and *STC1* (Knierim et al., 2016). And loss of function mutations was detected to be associated with a type of SMA (MIM: 253300; Knierim et al., 2016). Pathogenic variants in *ASCC1* downregulated genes associated with neurogenesis, neuronal migration, and pathfinding, as well as with bone development (Knierim et al., 2016). So far, a total of eleven patients from six independent families had been reported to be similarly affected by this perinatal lethal condition. Five variants including one highly recurrent frameshift variant (c.157dup (p.Glu53Glyfs*19) accounted for most of the pathogenic variants. Since this severe neuromuscular disorder caused by biallelic LOF variants in *ASCC1* is a relative new disease‐gene relationship and only a limited number of patient/pathogenic variants had been reported, we performed gene curation following the GlinGen gene curation protocol (https://clinicalgenome.org/site/assets/files/3907/gene-disease_validity_standard_operating_procedures_version_7-1.pdf). Based on previously published cases and this Chinese family, the total clinical validity score reaches 14 (10 points from genetic evidence and 4 points from experimental evidence), thus sufficient evidence supports a "Strong" gene‐disease relationship. Now 3 years had passed since the first reported cases (Knierim et al., 2016) and all affected individuals exhibited similar phenotypes and prognosis, overall, a "Definitive" level of disease‐gene relationship can be concluded. We reported the third case with a compound heterozygous variant. In the previously reported 11 cases, nonsense or frameshift variants are generally at the homozygous state and only one patient carried a compound heterozygous variant with the exons deletion (Giuffrida et al., 2019). Our patient is the second case carrying a similar compound heterozygous variant in *ASCC1*. The report of the two affected individuals from this Chinese family extended the mutational spectrum and provided further evidence that this condition also occurs in out populations.

**Figure 3:**
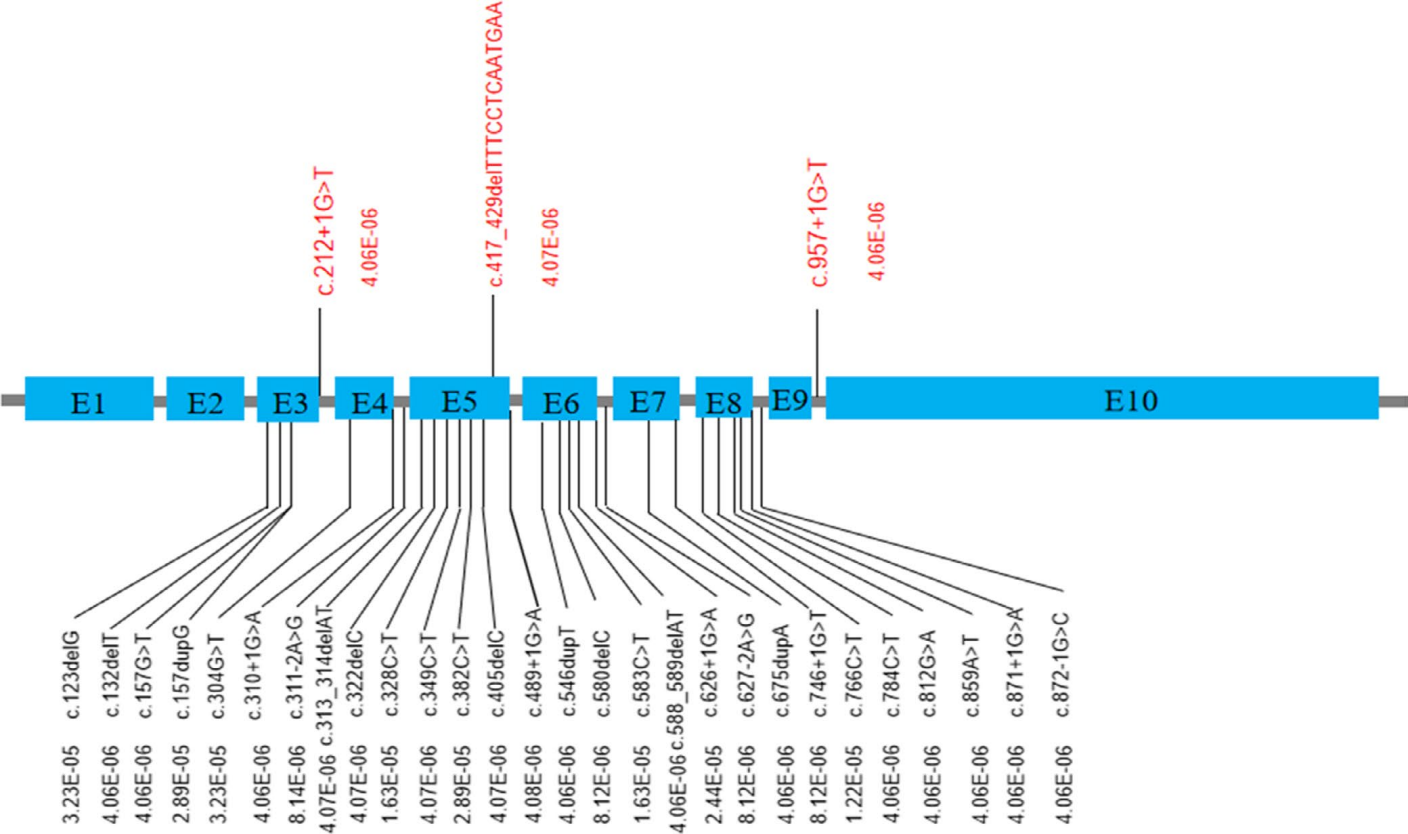
Schematic representation of exons and introns of *ASCC1* with the heterozygous mutations identified in East Asian population (red) and in the global population (black)

Loss of function variants in *ASCC1* are overall rare and never found to be homozygous in gnomAD (http://gnomad.broadinstitute.org/) except for the p.Ser78* nonsense variant which is located in an alternatively spliced exon and are believed to be not clinically relevant. There are 31 heterozygous LOF variants (Figure 3; should be at least likely pathogenic since both PVS1 and PM2 apply) and the overall pathogenic allele carrier rate is 0.0003, thus the estimated disease incidence would be in the order of 9/40,000,000. Only three LOF variants were found in East Asian population, including two splice donor mutations and one frameshift mutation, the carrier AF was lower than 0.0001, thus the disease incidence is expected to be lower in Asian. The mutation spectrum also different from population to population. Pathogenic variants from the general population are found to be concentrated in exons 3, 5, and 6, and it is consistent with the distribution of pathogenic variants detected in patients (Figure 2d), indicated that these 3 exons are more clinically relevant.

The clinical presentations of all reported patients are summarized in Table 2. This condition is mainly characterized by prenatal symptoms including decreased fetal movement, hydramnios, premature delivery, multiple fetal long bone fractures, arthrogryposis multiplex congenita, and stiffness and neonatal symptoms such as severe hypotonia, absence of deep tendon reflex, muscle weakness, muscle fiber abnormalities; abnormal cerebral cortical cerebral gyrus (Böhm et al., 2019; Giuffrida et al., 2019; Knierim et al., 2016; Oliveira et al., 2017). Our patient presented with hypotonia, congenital multiple deformity, neonatal respiratory distress, talipes equinovarus, and humeral fractures, which are all major features of this condition. Interestingly, our patient also exhibited additional features not previously reported including cyanosis, ecchymoses, pneumonia, and cryptorchidism. In addition, his prothrombin time, activated partial thromboplastin time, and thrombin time were significantly elevated (19.0, 76.3, and 26.5 s, respectively), indicating coagulation abnormality. These novel features may be added to our more complete understanding of this condition and can be used for differential diagnosis with other with SMA‐related disorders.

In conclusion, we reported the first Chinese *ASCC1* case with two novel pathogenic variants (the third compound heterozygous case). While the main clinical presentation of our patient is consistent with those of previously reported, novel phenotypes were also noticed. Although rare, this condition has a poor prognosis and can be missed due to a neonatal lethal outcome. The current evidence supports a definitive gene‐disease relationship. Additional cases are expected even in non‐consanguineous populations.

## CONFLICT OF INTEREST

The authors declare no conflict of interest.
